# Supplementary material for: Are carbon emissions trading and green financial instruments synergistic? -Comprehensive quantitative research based on content analysis
Source: PLoS One. 2024 Mar 7;19(3):e0298601. doi: 10.1371/journal.pone.0298601 (PMC10919601; doi:10.1371/journal.pone.0298601)
Supplement: S1 Data — (ZIP) [file pone.0298601.s001.zip › Data/地级市Z_F工作报告.zip!/║╙─╧/σo╤⌠╩╨/└┤╫╘╣1⁄2╓┌║┼í╛┬φ┐╦╩2╛▌═°í┐_2019.docx]

各位代表：

现在，我代表市人民政府，向大会报告工作，请予审议，并请各位政协委员和其他列席人士提出意见。

一、2018年工作回顾

2018年，是濮阳转型攻坚、爬坡过坎、向高质量发展迈进的一年，也是濮阳发展历程中极不平凡的一年。面对严峻复杂的经济形势和艰巨繁重的改革发展任务，在省委、省政府和市委的坚强领导下，全市上下以习近平新时代中国特色社会主义思想为指导，深入贯彻党的十九大精神和习近平总书记视察指导河南时的重要讲话精神，全面落实王国生书记、陈润儿省长莅濮调研工作要求，坚持稳中求进工作总基调，践行新发展理念，立足实际打好“四张牌”，聚焦聚力三大攻坚战，致力转型发展、创新发展，补短板、夯基础、创优势，经济持续健康发展，各项事业全面进步，社会大局和谐稳定。

2018年，我们统筹稳增长、促改革、调结构、惠民生、防风险，经济运行稳中有进，质量效益持续提升。全市生产总值完成1654.5亿元，增长5.8%。规模以上工业增加值增长6%。固定资产投资增长10%，高于全省1.9个百分点。一般公共预算收入完成91.7亿元，增长13%，高于全省2.5个百分点，税收占比73.9%，高于全省3.3个百分点。工业用电量增长14.2%，居全省第1位。各项贷款余额862.9亿元，增长15.9%，居全省第3位。居民人均可支配收入19802元，增长8.8%，高于全省0.3个百分点。常住人口城镇化率达到45.3%，提高1.6个百分点，增幅高于全省平均水平。

2018年，我们同心协力、苦干实干、奋力拼搏，为中原更加出彩增添了靓丽的濮阳色彩。巩固提升全国文明城市创建成果、顺利通过年度测评，蝉联国家卫生城市，成功创建国家森林城市，濮阳再添一块金字招牌。农村改革入围全国“改革开放40年地方改革创新40案例”。荣获全国人民调解工作先进集体、人民网网民留言办理工作先进单位。我市被确定为国家级社会管理和公共服务综合标准化试点。项目建设、招商引资、安全生产、食品药品安全、棚户区改造、农民工返乡创业、社会治安综合治理等工作走在了全省前列。

（一）产业转型升级成效明显。以供给侧结构性改革为主线，做强工业，做大服务业，做优农业，更加注重了质量效益和结构优化。

工业提质增效。突出“三大”“三专”，以“三大改造”为抓手，以项目建设为支撑，新旧动能加快转换。65平方公里新型化工基地总体规划获省批复，化工产业步入转型发展快车道。“一基五链三集群”产业体系已具雏形，丰利石化产值突破80亿元，龙德洋均苯四甲酸酐等10个项目建成投产，盛通聚源聚碳酸酯等24个项目加快建设。化工产业销售收入增长10.9%。实行化工企业“红黄蓝绿”标识管理，关停5家重污染企业，惠成电子被认定为国家级绿色工厂。工业园区绿色涂料产业园建设初见成效，开发区成功创建国家资源循环利用基地。推动装备制造、食品加工、现代家居、羽绒制品等产业提质发展，实施94个技改项目，66个项目竣工投产。实施58个智能化改造项目，培育20家智能车间、8家智能工厂。实施23个绿色化改造项目，9个项目建成投产。生物基材料产业产能达到50万吨，与河南机场集团、美团等加强战略合作，市场份额不断扩大。培育发展新产业，德力西智能电器一期建成投产，天能（濮阳）循环经济产业园四期扩能完成，销售收入突破60亿元。

服务业势头良好。金融业迈出新步伐，3家农信社成功改制农商行，银行业金融机构达到16家，中原农险等4家保险机构入驻濮阳，中航证券在濮设立分支机构获批筹建。物流业取得新成效，河南万通陆港电商物流中心等7个项目加快建设。货运周转量214.5亿吨公里，增长10.4%。文化旅游业成为消费热点，华龙区东北庄特色杂技文化园区、示范区班家耕读小镇等文旅项目扩容提质，成功举办首届中国·濮阳杂技文化艺术节，单拐冀鲁豫边区抗日根据地旧址成功创建国家4A级景区。旅游业收入30亿元，增长30%。文化产业增加值60.8亿元，增长6%。电子商务蓬勃发展，认定备案60家电商企业，电子商务交易额355亿元，增长18.3%。跨境电商企业达到120家，实现进出口3.8亿美元，增长20.6%。

农业质效双升。建成21万亩高标准粮田，优质农作物发展到286.7万亩，粮食总产287.1万吨。建设提升75个现代设施农业园区。培育了特色杂粮、食用菌、节水莲藕等一批地理标志农产品品牌和原产地保护基地。农民专业合作社、家庭农场、市级以上农业产业化龙头企业分别达到3207个、336个、228个。培育发展10大特色产业集群，牧原生猪养殖、新疆花麒奶业等全产业链项目综合效益不断提升，全市农产品加工业销售收入、利润分别达到448亿元、55.2亿元。

（二）基础能力建设实现突破。盯牢做实打基础、利长远的大事要事，狠抓项目建设，濮阳发展新优势不断蓄积。

现代交通体系加快构建。郑濮济高铁濮阳段建设全面铺开，濮阳人的高铁梦正成为现实。南林高速与德上高速实现互联互通。范台梁高速完成工程量的75%。濮阳至阳新、濮阳至卫辉两条高速和G240、S304两座黄河大桥全面开工在即。改建了53.3公里干线公路、764公里农村公路，范县、南乐县分别成功创建国家、省级“四好农村路”示范县。人民群众出行更加便捷。

资源能源保障逐步强化。鄂安沧输气管道濮阳支干线全线连通，文23战略储气库一期工程达到注气条件，库容300亿立方米的国家天然气战略储气库群初具规模。日濮洛输油管道开工建设并在濮阳预留门站。豫能2×60万千瓦2号机组列为全省煤电应急调峰储备电源启动运行，濮润2×2.5万千瓦机组、塔铺至卫都500千伏线路加快建设。引黄入冀补淀工程正式通水。南水北调濮阳县、南乐县配套工程完工，115万群众喝上丹江水，南水北调供水乡镇受益规模居全省首位。

重点项目建设成效显著。落实市级领导分包、周例会、联审联批、督导通报等制度，举全市之力，开展百日攻坚行动，348个省市重点项目提前完成年度任务，其中77个省重点项目超额完成省定目标，万嘉360广场等72个项目建成投用。综合排名位居全省第一方阵。

（三）乡村振兴战略扎实推进。以“五大振兴”为路径，以脱贫攻坚为重点，乡村振兴开局良好。

脱贫攻坚有力有效。坚持精准方略，聚焦深度贫困，突出抓好产业扶贫、金融扶贫、转移就业。拓宽增收渠道，重点扶持69家龙头企业和新型农业经营主体，发放扶贫小额贷款17.1亿元，新增户贷率48.4%、居全省第4位。贫困家庭劳动力转移就业率85%。6.6万贫困人口实现脱贫，濮阳县脱贫摘帽成功在望。黄河滩区居民迁建第二批试点安置区达到入住条件，习城安置区、东桥安置区714户群众喜迁新居；三年规划12个安置区加快建设，范县县城安置区建设进度居全省第一。

乡村振兴有序实施。清丰县被确定为省乡村振兴示范县，庆祖、张果屯等7个乡镇被确定为示范乡镇。开展农村人居环境整治，完善城乡环卫一体化运营机制，五个县全部通过农村生活垃圾处置省级验收。建成34座乡镇垃圾中转站、146座集镇公厕、3个污水处理厂。实施100个美丽乡村示范村、26个精品村建设。中央电视台对我市濮渠路、阳子线美丽乡村精品线路进行了报道。建成1989个基层综合文化服务中心，开展“舞台艺术送基层”等活动，农村文化生活更加丰富。

（四）百城提质深入开展。将百城提质与文明城市创建有机结合，把握“四个统筹”，做好“四篇文章”，突出抓好“四治”，由注重外延扩张向注重内涵提质转变。

城市综合承载力不断增强。第四轮城乡总体规划通过评审。市中心城区，实施175个城建项目，完成投资80.7亿元。茂名路改造竣工投用，106国道、金堤路北延等道路加快建设。高标准实施引黄灌溉调节水库岸线综合提升工程，新增绿地面积24.8万平方米，绿化覆盖率40.7%，人均公园绿地面积14.8平方米。市第二污水处理厂二期工程调试运行。实施31个海绵城市建设项目，6个项目竣工，覆盖面积11.5平方公里。礼尚、谢庄农贸市场完成改造，凯旋城市场建成投用。县中心城区，开工建设238个城建项目，完成投资216.3亿元。新建改造64条91.7公里市政道路，铺设59.3公里供水管网、104公里雨污管网、142公里供暖管网。濮阳县龙文化主题公园、南乐县森林公园基本建成。全市实施棚改项目56个、55506套，开工率101.5%、建成率130%，居全省前列。房地产开发投资194亿元，增长28.8%，居全省第3位。

小城镇建设步伐加快。因地制宜、分类指导、循序渐进，着力提升基础设施，完善公共服务，发展特色产业，建设生态宜居、乡风文明、管理规范、富有活力的小城镇。文留、柳屯、濮城等荣获全国文明村镇。培育马辛庄民俗小镇、侯庙绿色光伏小镇、韩张北方水乡小镇等16个特色小城镇。

（五）生态环境持续改善。践行“绿水青山就是金山银山”理念，坚持治理、保护、建设并重，生态文明建设取得阶段性成效。

污染防治全面发力。开展“环保宣传进企业下工地”活动，建立综合督导、联合执法、明查暗访、考核排名、约谈问责机制。整治取缔12家“散乱污”企业，落实工地施工“六个百分之百”要求，加强重型运输车辆排放管控，实施重要时段单双号限行、公交车免费。挥发性有机物治理工作走在全省前列。新增天然气用户8万户、供暖面积588万平方米，全市天然气普及率、市城区集中供热普及率达到71.3%、90.8%，均居全省第1位，我市被列入国家北方地区清洁取暖试点城市，完成“双替代”11.2万户。PM10、PM2.5、优良天数实现“两降一增”，位居全省前列。在京津冀“2+26”城市中，我市年度空气质量综合指数排名第8位。全面推行河（湖）长制，完成新老马颊河、濮水河城区段截污治污、清淤疏浚和黑臭水体整治，河流出境断面水质全部达标。土壤环境质量保持稳定。环境质量改善率和群众满意度实现双提升。

生态建设富有成效。以实施国储林项目为抓手，加强生态廊道、农田林网、村庄庭院绿化建设，全市植树造林1000万株、9.3万亩，森林覆盖率30%以上。建设了金堤河湿地、马颊河湿地等生态工程，全市湿地面积达到20万亩。

（六）发展动能加快转换。坚持以改革破瓶颈、以开放添活力、以创新增动力，发展动能持续释放。

重点领域改革全面深化。“放管服”改革成效明显，投资项目审批事项由91项减至48项，电子政务外网实现市、县（区）、乡镇政务部门全覆盖，“最多跑一次”网上可办率、一网通办率达到100%。农村综合改革全省领先，集体土地所有权和使用权确权登记发证到户率91.6%，居全省第1位；99.6%的农村承包地完成确权，发放产权类抵押贷款5935笔、4.31亿元；我市被确定为全国农村集体产权制度改革整体推进试点市，农村集体资产清产核资全面完成，87.7%的行政村成立了集体经济组织，70%的行政村成立了股份经济合作社。国企改革稳妥推进，48家“僵尸企业”处置工作完成省定目标，驻濮央企“四供一业”完成移交8.7万户、涉及8000名从业人员。油地合作持续深化，共同推进了战略储气库群、中原石化原料结构调整等重大项目。投融资方式不断创新，市投资集团联合郑州银行，成功发行首期境外1亿美元债券；多渠道融资40亿元，争取债券资金50.5亿元。锐实达石油设备等51家企业在中原股权交易中心挂牌上市。综合执法、医疗卫生等改革取得阶段性成效。

开放水平大幅提升。扩大招商引资，新签约亿元以上项目323个，新开工268个。施耐德、东旭蓝天等17家世界500强企业和上市公司落户濮阳。实际利用省外资金244亿元，增长6.6%，居全省第2位。实际利用境外资金6.6亿美元，增长3.3%。完善开放平台，濮阳海关获准设立，国检濮阳办事处、海关窗口全面开展业务，终结了我市企业异地报关、报检的历史。公共型保税仓库建成投用，填补了我市无海关保税监管场所的空白。濮阳至天津港铁海联运班列开辟了河南海上丝绸之路新通道。功能聚合物及复合材料、羽绒2个国家级外贸转型升级基地获批。丰利石化获得原油非国营贸易进口资格和222万吨原油进口指标。完成货物贸易进出口54.6亿元，增长36.7%，居全省第5位。

创新驱动深入实施。新培育9家国家高新技术企业、10家市级创新龙头企业、18家科技小巨人企业、10家省市级节能减排科技创新示范企业。新建15家市级以上工程技术研究中心、7家市级重点实验室。新建河南省君恒生物科技院士工作站，全市院士工作站达到5家。加强产学研合作，市政府与中科院河南创新中心建立战略合作关系，绿探集团与哈工大合作成立产业技术研究院，成功创建国家企业技术中心；462家企业与高校、科研院所开展项目合作598项。柔性引进7名院士及团队成员、321名高端急需紧缺人才。天健生物董事长苏庆丰入选“中原千人计划”中原科技创业领军人才。万人有效发明专利拥有量1.89件，增长4.4%。成功举办2018年中国（濮阳）石化产业融合发展大会。

营商环境持续优化。出台《解决当前营商环境突出问题的若干措施》等重磅文件，建设诚信社会，构建“亲”“清”政商关系，保护企业家权益，强化要素保障。据2018年上半年河南省企业活跃度研究报告，我市企业活跃度指数全省第一。全市新登记各类企业、实有市场主体分别增长12.9%、12.5%。民间投资增长11.9%，高于全市投资增速1.9个百分点。非公有制经济增加值占生产总值的67.5%，对经济增长的贡献率73.5%，成为经济发展的重要支撑。落实惠企政策，为企业降本减负13.1亿元。

（七）民生福祉不断提升。加大民生投入，民生支出244.7亿元，占一般公共预算支出的79%，高于全省1.8个百分点。省定民生实事全部完成，“雪亮工程”、教育基础设施建设2件市定民生实事加快推进，人民群众获得感、幸福感、安全感不断增强。

就业社保水平稳步提高。城镇新增就业5.17万人，完成省定目标的134.4%，城镇登记失业率2.26%，低于全省平均水平0.76个百分点。南乐县、清丰县、台前县被列入全国新型城镇化支持农民工返乡创业试点，全市4.3万农民工返乡创业，创办4.2万个经营主体，带动22万人就业。企业退休人员养老金实现“十四连增”。城乡居民基础养老金最低标准提高到100元。居民医保财政补助标准提高到490元，困难群众大病补充医疗保险参保登记率达到100%，17种治疗重特大疾病的特效靶向药、仿制药纳入医保范围，实现跨省异地就医直接结算定点医疗机构县级全覆盖。7万名残疾人获得生活补贴和护理补贴，残疾人综合服务设施在全省率先实现全覆盖。

社会事业全面发展。编制完成市城区中小学布局规划，市城区新建4所学校，改建6所；县城新建18所学校，改建2所，全市新增3.3万个学位。改善52所农村薄弱学校，台前县成功创建全国义务教育发展基本均衡县。新建6所幼儿园，改建30所。高考一本上线率高于全省平均水平5.3个百分点。高等教育在校生突破2万人。改建25个医疗卫生机构，新增4323张床位，建成4家医养结合机构。在全省首发电子健康卡，实现跨区域、跨医疗机构健康服务“一卡通”。妇女两癌筛查、新生儿两病筛查完成省定目标。完善市图书馆新馆、市工人文化宫功能，新增10个社区图书馆。建设了马颊河体育公园，建成26处便民健身场所。35名杂技学员入选国家滑雪队，自由式小轮车国家队训练基地落户我市。中国清丰极限运动基地基本建成，成为国内最大的综合性极限运动基地。

社会治理更加高效。防范经济发展、自然灾害、公共事件等重点领域风险，守住了不发生系统性、区域性风险底线。安全生产监管水平不断提高，建成国内最大的国家危险化学品应急救援（实训）基地。食品药品安全形势持续稳定。深入开展扫黑除恶专项斗争，依法做好信访工作，社会大局保持稳定。

国防动员教育及双拥工作扎实开展，驻濮解放军和武警部队、消防救援队伍在支持地方发展、维护社会稳定方面做出了积极贡献。统计、民族宗教、发展研究、外事侨务、机关事务、驻外联络、公积金管理、气象、防震减灾、人防、史志、档案等工作都取得了新成绩。

依法接受人大监督，自觉接受政协民主监督和社会监督，认真办理人大代表建议和政协提案，广泛听取民主党派、工商联、无党派人士意见，支持工会、共青团、妇联等人民团体开展工作。加强法治政府建设，推进依法行政，强化监察、审计监督，持续纠正“四风”，政府自身建设和治理能力不断提升。

各位代表！回首过去的一年，看似平常，实则不易，栉风沐雨，砥砺奋进。这一年，我们牢牢把握高质量发展根本要求，践行新发展理念，深化供给侧结构性改革，转型发展、创新发展有力有效、后劲十足；这一年，我们聚焦聚力三大攻坚战，尽锐出击、靶向施策，攻克了多重堡垒、守住了风险底线；这一年，我们致力推动边缘变前沿，加快构建基础支撑体系，不断完善开放载体平台，目标明确、步履铿锵；这一年，我们持续深化重点领域改革，切实优化营商环境，形成了一批改革亮点，推出了一批叫响全省全国的“濮阳经验”“濮阳模式”。我们在前进道路上迈出的每一步，取得的每一点成绩，都源于省委、省政府和市委的坚强领导，源于市人大、市政协的监督支持，源于全市人民的拼搏奋斗。在此，我代表市政府，向全市工人、农民、知识分子、干部、人民解放军指战员、武警官兵、公安民警、消防救援队伍指战员及所有关心、支持濮阳发展的各界人士、各界朋友致以崇高的敬意和衷心的感谢！

我们也清醒地认识到，一些工作做得还不到位，经济社会发展中还面临不少困难和问题：一是转型发展任务艰巨。主导产业、传统产业层次不高，现代服务业占比偏低，高技术产业、战略性新兴产业发展滞后。二是创新驱动能力不强。人才、机构、平台等创新资源匮乏，研发投入强度低于全国、全省平均水平。三是三大攻坚战任务繁重。风险隐患依然存在，精准脱贫攻坚到了啃“硬骨头”的关键时期，污染防治攻坚工作形势十分严峻。四是交通瓶颈尚未根本破解，城市功能还需完善，教育、医疗等公共服务水平还需提高，城市承载力和辐射力还需增强。五是干部作风能力建设还需进一步加强提升。解决这些问题，是破解濮阳高质量发展瓶颈制约的关键，我们将加倍努力，把各项工作做得更实更好。

二、2019年工作总体要求和主要目标

2019年是新中国成立70周年，是全面建成小康社会的关键之年，是推动濮阳高质量发展的攻坚之年，做好今年政府各项工作至关重要。综合来看，尽管外部环境复杂严峻，经济面临下行压力，但我市仍面临前所未有的机遇。中央对重要战略机遇期的判断，提振了我们推动高质量发展的信心；省委、省政府对濮阳的关心支持，鼓舞了我们推动高质量发展的士气；濮阳日益完善的发展基础条件，增强了我们推动高质量发展的底气。濮阳发展未来可期、大有可为。我们要立足改革开放40年历史巨变，顺应发展长期大势；立足国际国内两个大局，审视当前发展形势；立足区域协同发展格局，把握竞合发展态势，抢抓战机，争得先机，开创生机，牢牢把握高质量发展主动权。

今年政府工作的总体思路是：坚持以习近平新时代中国特色社会主义思想为指导，全面贯彻党的十九大和十九届二中、三中全会及中央经济工作会议、省委十届八次全会、市委七届九次全会精神，持续落实习近平总书记视察指导河南时的重要讲话，统筹推进“五位一体”总体布局，协调推进“四个全面”战略布局，坚持稳中求进工作总基调，坚持新发展理念，坚持推动高质量发展，坚持以供给侧结构性改革为主线，坚持深化市场化改革、扩大高水平开放，贯彻巩固、增强、提升、畅通的方针，深入开展三大攻坚战，持续推进“四个着力”、打好“四张牌”，统筹推进稳增长、促改革、调结构、惠民生、防风险、保稳定工作，保持经济运行在合理区间，进一步稳就业、稳金融、稳外贸、稳外资、稳投资、稳预期，提振市场信心，增强人民群众获得感、幸福感、安全感，保持经济持续健康发展和社会大局稳定，加快由区域边缘走向中原城市群改革开放前沿，为全面建成小康社会收官打下决定性基础，为中原更加出彩增添更靓丽的濮阳色彩，以优异成绩庆祝中华人民共和国成立70周年。

今年经济社会发展主要预期目标是：生产总值增长7%以上，力争达到7.5%，全员劳动生产率增长7%，规模以上工业增加值增长7.5%以上，一般公共预算收入增长8%以上，税收占一般公共预算收入比重72%以上，固定资产投资增长8.5%，社会消费品零售总额增长10%以上，居民消费价格涨幅3%以内，经济结构进一步优化，质量效益明显提升。创新发展取得新成果，研究与试验发展经费投入强度1%，高技术产业增加值、战略性新兴产业增加值均增长10%以上。协调发展取得新成效，常住人口城镇化率提高1.65个百分点，人均预期寿命持续提高，九年义务教育巩固率95%以上，医疗卫生、文化、体育等社会事业全面进步。绿色发展取得新提升，单位生产总值能耗下降3%，主要污染物排放量完成省定目标。开放发展取得新突破，货物进出口增长8%，营商环境进一步优化。共享发展取得新进步，居民人均可支配收入增长8%以上，4.13万贫困人口稳定脱贫，范县、台前县脱贫摘帽。城镇登记失业率控制在4%以内。以上预期目标，更加凸显了新发展理念和高质量发展要求，全面贯彻了中央、省稳增长工作要求，充分考虑了濮阳的产业基础、项目建设、投资拉动等支撑因素，既契合濮阳实际，科学可行，又跳起来摘桃子，积极作为，努力争取更好的结果。

做好2019年政府工作，我们一定强化“四个意识”，坚定“四个自信”，坚决做到“两个维护”，确保中央、省和市委决策部署落地生效。坚持稳定经济增长不动摇，不断夯实高质量发展基础。把稳增长作为首要任务，咬定目标、多措并举，保持较快的发展速度和强劲的发展势头，为高质量发展创造条件、赢得空间。推进产业转型不放松，切实强化高质量发展支撑。把促转型作为战略重点，突出“三大”“三专”，改造提升传统产业，培育壮大新兴产业，做大做优服务业，加快质量变革、效率变革、动力变革，构建现代产业体系。打好重点战役不懈怠，持续补齐高质量发展短板。坚持问题导向、结果导向，精准施策，合力攻坚，确保脱贫实效，切实改善生态环境质量，为全面建成小康社会奠定坚实基础。深化改革开放不停步，着力增强高质量发展动力。保持改革韧劲、增强改革定力，深化重点领域改革，破除体制机制障碍，充分激发市场活力，增强微观主体动力；放宽视野、积极作为，主动融入“一带一路”、中原城市群、京津冀协同发展等重大战略，强化与周边城市的合作联动，加大招商引资力度，形成高水平开放新格局。紧盯风险防控不回避，努力营造良好高质量发展环境。树立底线思维，紧盯经济发展、政治安全、社会稳定、自然灾害、公共事件、安全生产六大领域风险，切实解决可能影响高质量发展的突出问题，坚决守住不发生系统性区域性风险的底线。增进民生福祉不松劲，让人民共享高质量发展成果。坚持把人民放在心中最高位置，顺应民心、尊重民意、关注民情，集中力量做好普惠性、基础性、兜底性民生建设，解决好群众关心的热点、难点问题，让人民群众的生活芝麻开花节节高、一年更比一年强。

三、2019年工作安排

围绕今年的预期目标，重点抓好七个方面工作。

（一）促进经济稳定增长，保持稳中有进发展态势。落实中央“六稳”要求，保运行、扩投资、促消费，确保经济平稳健康增长。

贯彻“八字方针”。深化供给侧结构性改革，巩固“去降补”成果，持续推进“僵尸企业”后续处置，做好债务化解、破产审理、资产处置、职工安置等工作，6月底前完成后续处置任务；推动水泥、煤化工产业以“减量、延链、提质”为重点，化解过剩产能、退出低效产能。增强微观主体活力，推动相关改革走深走实，激发企业内生动力，释放企业发展潜力。提升产业链发展水平，抓好产业技改扩能，推进产业集群融合，增强产业竞争优势。畅通经济循环系统，完善市场体系，改善金融服务，发展现代物流，畅通生产、流通、分配、消费循环，提升经济发展质量效益。

壮大实体经济。落实减税降费政策，降低企业成本，让企业有实实在在的获得感。引导支持企业顺应消费需求，调整产品结构，提高供给质量。强力实施“三个计划”。实施大企业培育三年行动计划，加快技术创新、管理创新、商业模式创新，培育行业领军企业，推动优势产业扩规模、上水平、增效益。今年力争主营业务收入丰利石化达到100亿元，濮阳天能达到80亿元，中原石化、中原大化达到50亿元，盛源集团、宏业控股达到30亿元，龙丰纸业达到25亿元。实施中小企业成长计划，重点培育迈奇化学、海林装备、凯利来食品等50家高成长性企业，梯次培育，滚动发展。支持惠成电子、鹏鑫化工等企业成为细分行业的领跑者、特色产品的全国乃至世界“单项冠军”。大力发展非公有制经济，支持民营企业制度创新、科技创新、产品创新，促进二次创业和转型发展。实施企业家培育计划，组织领军型企业家和新生代企业家到知名高校进行高端培训，重点培训80名企业高管，提升企业家队伍素质。

优化企业服务。坚持市级领导分包重点企业、企业服务日、企业服务直通车等制度，帮助企业纾困解难。破解融资难题，用好民企债券融资支持计划，推动政府基金、产业基金与企业股权合作；完善金融机构考核体系，健全尽职免责和容错机制；引导企业规范经营行为，增强管理和融资能力，让金融服务回归实体经济。支持市投资集团等市属国有企业做大做强。坚持周调度、月分析，加强监测调控，确保经济运行在合理区间。

扩大有效投资。发挥投资拉动经济增长的关键作用，实施270个重点项目，年度完成投资956.6亿元。其中，131个新建项目，139个续建项目，87个项目建成投用。突出重点项目、重大专项，细化任务，建立月台账、季台账，夯实责任，明确节点，强力推进。加强项目储备，精准谋划一批项目，争取纳入国家、省计划盘子。稳定房地产投资，促进房地产健康发展。

发展现代服务业。物流业，谋划实施绿色涂料区域交易中心、石化能源物流园等项目，加快建设木伦河冷链物流园，引进浙江传化物流港，建设物流业大数据平台，争取京东、顺丰等企业在濮设立区域总部、服务中心、集配中心、云仓等。金融业，争取平安银行等股份制银行在濮设立分支机构，引进私募基金，建设智慧金融平台。支持企业利用金融创新产品融资发展。争取1到2家企业在境内外上市，一批企业在新三板和中原股权交易中心挂牌。信息服务业，加快大数据智慧生态产业园建设，加强与浪潮、南威软件等企业合作，建设区域性数据中心，入驻企业100家以上。启动工业云二期平台建设，培育扶持华阳科技、大盛电子等本地信息服务企业做大做强。加快中科曙光云计算中心建设，打造全国石化工业大数据应用示范基地。文化旅游业，重点建设中华龙源景区、顿丘影视文化小镇等项目。组建旅游发展集团，建设旅游集散中心，打造精品旅游线路，推介龙文化、杂技文化等特色产品，叫响国家历史文化名城品牌，打造华夏历史文明传承创新区。促进杂技与体育、旅游跨界融合，深化与国家体育总局战略合作，承办首届中国极限运动大会；争取承办2019年中国杂技艺术节。康养业，实施协和中医院颐养综合体、华龙区健康养老等22个项目，引进健康养老知名企业，建设区域性康养中心。商贸服务业，实施油田商贸中心改造、新天龙市场、滨河商业街等重点项目。加强县城商务中心区和特色商业街区建设。发展电子商务，建设南乐县电子商务仓储中心等项目，新认定备案30家电商企业。新培育2家跨境电商产业园，提升濮阳跨境电子商务产业园运营水平。

（二）加快产业转型，建设全国重要的新型化工基地。坚持工业强市战略，持续抓好“三大”“三专”产业发展，实施包括化工产业项目、现代家居产业项目、羽绒及服饰加工产业项目、生物基材料产业项目的十大工业重点建设工程，做到外部引进与内部培育双向发力，技改扩能和新上项目齐头并进，龙头带动和企业配套集群发展，打造产业链，提升价值链，构建创新链，推动制造业高质量发展。

突出化工产业强基提升。围绕“一基五链三集群”，强基础、聚集资源，做大上游；延链条、深耕细作，做精下游。以7个化工产业重点建设工程为引领，加快构筑支撑点，带动化工产业向绿色化、循环化、智能化、高端化发展。开工建设投资40亿元的中原石化原料结构调整、投资40亿元的远东科技丙烷脱氢、投资30亿元的绿色新型涂料产业集群、投资15亿元的朗润新材料橡胶助剂清洁化生产等项目；建成投资24亿元的君恒实业重蜡加氢、投资21亿元的盛通聚源聚碳酸酯、投资10亿元的德容新材料循环经济产业园等项目，实现试产和达产。

加快传统产业改造提升。现代家居产业方面，实施广东、浙江商会家居产业集群项目重点建设工程，加快打造中部地区实木家具基地。羽绒及服饰制品产业方面，加快推进羽绒及服装加工产业集群项目重点建设工程，做好“互联网+羽绒”文章，提高产品知名度和影响力。深化工业“三大改造”，实施95个重大技改项目，增强传统产业创新力；创建5家智能工厂、10家智能车间、2家绿色工厂，推进两化融合和智能制造。装备制造产业方面，推动中拓管道、锐驰高科等油气装备企业向“产品+服务”方向拓展。加快中宝元智能装备制造、星光农机制造等项目建设。

积极培育高技术产业和战略性新兴产业。敢于无中生有，善于有中生新，在新材料、新能源、节能环保等战略性新兴产业发展上力求突破。立足现有基础，加快生物基材料集群项目重点建设工程，壮大规模，拉长链条，开拓市场，增强集群影响力。推动施耐德、德力西深化战略合作，在濮建设中部地区智能电器制造基地。加强军民融合发展，依托华南重工建设中国兵器北方永磁电机濮阳基地。实施濮耐新型高温材料、展辰新材料等21个项目。谋划建设应急救援装备制造产业园。依托我市电子化学品产业优势，积极引进电子元器件、通信设备、智能终端等领域行业龙头和品牌企业，培育电子信息产业。

打造产业转型平台。把65平方公里新型化工基地作为转型发展主平台，建立高效工作机构和机制，制定统一的项目准入、规划、土地、招商、税收、融资等配套政策，为基地建设提供组织和政策保障。修改完成空间规划和水电气热等市政专项规划；编制完成化工新材料、专用化学品、功能化学品三大产业集群发展规划和物流仓储园区专项规划。实施道路、综合管廊、污水处理等29个基础设施项目，增强服务和承载能力。

（三）打好三大攻坚战，补齐发展短板。三大攻坚战到了攻城拔寨的总攻期，必须精准发力，苦干实干，决战决胜。

防范化解重大风险。做好政府性债务化解，依法规范政府举债行为，科学安排年度预算资金，有效盘活财政存量资金，把好融资关口，严控债务增量。防范化解金融风险，强化金融监管，化解金融机构不良贷款，开展互联网金融风险专项整治，严厉打击非法集资，营造良好金融生态。化解企业风险，加强排查，分类管控，稳妥化解。压实安全生产责任，全面推行双重预防体系建设，抓好源头管控治理，坚决防范遏制各类事故发生。增强防洪抗灾能力，防范自然灾害风险。妥善处理问题楼盘、征地拆迁、物业管理等历史遗留问题。健全应急管理体系，做好突发公共事件预防处置，防范社会风险。

狠抓精准脱贫。扎实推进“五个一批”，实施1651个扶贫项目，完成投资30.7亿元。突出产业扶贫，每个县（区）集中打造2到3个主导产业，培育一批扶贫龙头企业、专业合作社，巩固提升扶贫车间带贫能力，确保每个有劳动能力的贫困户有1项以上产业扶贫项目，每个预脱贫贫困户有2项以上增收措施。强化金融扶贫，发挥扶贫小额贷款作用，支持贫困户发展产业。实施“政银企”等金融扶贫项目，提高金融扶贫覆盖率和获贷率。做好电商扶贫，发展12家农村电商龙头企业，贫困乡镇电商覆盖率达到100%，贫困村覆盖率达到80%以上，吸纳5000名以上贫困人员就业。全面落实教育、健康、社保、安全饮水等扶贫政策。做好已脱贫群众跟踪服务，推动由稳定脱贫向逐步致富转变。

加强生态环境保护。打好蓝天保卫战，调整产业结构，优化化工产业布局，实施西减东加、退城入园，严格“红黄蓝绿”标识管理，分批次搬迁78家黄牌企业，整改36家蓝牌企业；调整能源、交通运输、用地结构，抓好扬尘、企业排放、机动车尾气专项治理。打好碧水保卫战，完成市县城区黑臭水体整治，强化饮用水水源地保护。打好净土保卫战，做好土壤污染防治与生态修复工作。做好中央、省环保督察反馈问题整改工作。实施国土绿化提速行动，以国储林项目为依托，开展冬春植树造林，植树1000万株、造林8万亩以上，沿黄河、金堤河、马颊河、引黄入冀补淀工程建设4大生态走廊，建设12个森林公园和湿地公园、73个森林乡村、10个森林驿站，与生态共生，与自然共融，打造天蓝地绿、水清气净、鸟语花香的美丽家园。

（四）强化基础支撑，厚植发展优势。努力打造豫东北门户枢纽城市，加快基础能力建设，促进区域融合发展，推动濮阳由区域边缘走向中原城市群改革开放前沿。

坚持交通先行。突出抓好交通基础设施“双十”工程。新建续建10个重点交通项目：完成郑濮济高铁濮阳段线下工程，开工建设濮阳高铁站和濮阳至豫鲁省界段；范台梁高速范县至台前段建成通车，加快推进濮阳至阳新、濮阳至卫辉高速公路建设；开工建设G240范县杨集、S304濮阳县白堽黄河大桥，复工建设G342台前孙口黄河大桥；实施G342、S214（东环）、S215公路改扩建项目。积极推进衡水至濮阳至潢川铁路，濮阳至安阳城际铁路，濮阳至菏泽城际铁路，德单高速台前段，濮卫高速东延段，阳新高速北延段，濮阳西北环（濮阳至豫北机场快速通道），范县至莘县公路，台前至阳谷公路，台前至东平公路等10个重点交通项目前期。加快构建与山东、雄安及周边路网外联内畅、便捷高效的现代交通体系。

汇聚资源能源。深化与中石化合作，加快建设国家天然气战略储气库群，力争参股中石化中原储气库有限公司。开工建设文23储气库二期工程，推进卫11、文13西、文24储气库前期工作。加快建设鄂安沧输气管线濮阳支干线输气门站，尽快调试投用；建成濮范台输气管线，建设中原和华北地区重要的天然气输配中心、交易中心。加快日濮洛输油管线建设。建成濮润背压热电联产项目，推进塔铺至卫都500千伏线路、石家庄至菏泽1000千伏线路濮阳段等工程，打造“双核三环”供电主网架。建设华能一期50万千瓦风电项目，建成南乐仓颉10万千瓦、台前华能4万千瓦风电项目，开工建设天润顿丘二期14万千瓦风电项目。

实施“四水同治”。出台水利发展总体规划、水资源综合规划，启动编制“四水同治”建设规划和专项规划。实施黄河下游涵闸改建、大型灌区续建配套与节水改造、城市供水调蓄池等九大“四水同治”工程，推进引黄能力提升、沟渠贯通生态治理等77个项目建设。编制完成马颊河综合治理规划，启动河道清淤工作，与聊城开展联动共治，做好区域水资源保护利用文章。加强地下水生态保护，封闭180眼城区自备井，做好节水型城市创建工作。落实河（湖）长制，加强河湖管理，开展“清乱占、清乱采、清乱堆、清乱建”专项行动，为高质量发展提供坚强水利保障。

（五）统筹城乡建设，促进协调发展。以百城提质和乡村振兴为抓手，加快新型城镇化进程，着力构建城乡融合发展新格局。

全面开展百城建设提质。实施中心城市带动战略，全面提升县城综合承载能力，走内涵式、高质量发展道路，做好“四篇文章”，抓好城市“四治”，实现城市规划提标、建设提质、管理提优。加快规划编制。启动国土空间总体利用规划编制；完成中心城区东南片区、东北片区等控制性详细规划，重点做好高铁片区、商务中心区城市设计，优化城市布局和形态；完成各县33项专项规划编制和重点片区的城市设计。完善基础设施。优化中心城区路网结构，推进市中心城区与濮阳县城区、清丰县城区、新型化工基地路网等基础设施的连接，加快一体化进程。打通振兴路、任丘路等“断头路”，改造提升五一路、文明路、南海路等老旧破损道路。开展微改造、微建设，基本完成城区易涝点治理。提升市政公用服务能力，开工建设第一污水处理厂扩容和餐厨垃圾处理项目，建成生活垃圾填埋场二期和焚烧发电项目，建设建筑垃圾堆放场。五个县城，实施好312个百城提质项目，加大路网、水气暖供应和垃圾污水处理等基础设施投入力度，推进县城服务保障能力提质、提速、提效。实施棚改攻坚行动。将2019年确定为中心城区棚改攻坚年，以市城区城中村改造为重点，推动城市人居环境改善、功能完善和品质提升。抓好第一批纳入省计划的7个棚改项目，年内完成投资24.7亿元。推进华龙区、开发区两个“五村整合”项目，加快胡村南街改造，启动京开大道南北入市口、胡村集三个片区旧城改造，完成文化路、黄河路大杂院等片区拆迁，利用三到五年时间完成中心城区棚改和提升任务。开展城市“四治”。完善“两级政府、三级管理、四级网络”城市管理机制，推动城市管理向城市治理转变。有效整治“两违”“六乱”、停车难等城市突出问题，提升城市精细化管理水平。搞好城市经营。强化土地收储，持续开展批而未用土地专项治理，优化资源配置，筹集建设资金，为百城建设提质提供坚强支撑，探索自我积累、自我增值、以城促产、循环发展的新路子。提升城市品位。做足绿、做活水、融入文、扮靓城，建设城市绿系水系，打造具有北方特色的园林城市；注入特色文化符号，延续城市历史文脉；实施城市夜景亮化工程，彰显城市魅力；完善教育、医疗等公共服务功能，建设宜居宜业之城。

大力实施乡村振兴战略。打造绿色高效农业创新发展示范区。抓好310.8万亩粮食生产功能区、20万亩重要农产品生产保护区建设，稳定粮食综合生产能力。调整优化农业结构，持续推进“六优四化”，做大优质优势农产品基地，种植面积达到294.5万亩。以“粮头食尾”“农头工尾”为抓手，大力发展农产品精深加工，提高农副产品增加值。实施百亿级四大农业特色产业集群培育工程，培育龙头，壮大规模，塑造品牌，提质增效，做大做强以中粮集团、豫粮集团、家家宜米业、伍钰泉面业等为龙头的粮食加工产业集群，产值达到30亿元以上；以汇源羊业、禾丰肉鸡、木伦河冷饮等为龙头的畜牧养殖加工产业集群，产值达到45亿元以上；以桃园建民、道德金等为龙头的副食品加工产业集群，产值达到20亿元以上；以龙丰实业、龙乡红等为龙头的食用菌产业集群，产值达到15亿元以上。围绕莲藕、辣椒、泥鳅、小龙虾等特色农业，再培育一批农业产业化龙头企业。坚持质量兴农，加强质量安全监管和品牌建设。大力发展农业生产性服务业和乡村生活性服务业，促进农业与旅游、教育、文化、康养等产业深度融合，提升农业综合效益。培育家庭农场、农民合作社等新型经营主体，把小农户生产引入现代农业发展大格局。支持农民返乡创业，鼓励各类人才到农村大显身手。深入实施农村人居环境整治。借鉴浙江“千村示范、万村整治”经验，治理农村“脏乱差”。编制各县区的村庄布局规划和清丰县、南乐县、7个省级示范乡镇的村庄建设规划。聚焦垃圾治理、污水处理、厕所革命等重点工作，推动垃圾处理标准化，实现农村生活垃圾治理全覆盖。建成17个乡镇污水处理厂及配套设施。新建120个美丽乡村示范村，高标准打造40个“四美乡村”。倡树文明乡风，提升乡村治理能力。巩固提升农村集体产权制度改革成果。推进农村集体经营性资产股份合作制改革，推动资源变资产、资金变股金、农民变股东。扩大“两权”抵押贷款覆盖面，开展农村集体资产和集体股权抵押贷款试点。创新运行机制，探索通过自主开发、参股农业产业化龙头企业、村企联手共建等多种形式，发展新型集体经济，增加村集体经济收入，让改革成果惠及农民群众。当好农村改革探路者，在全省全国形成可复制、可推广经验。

（六）深化改革开放，激发动力活力。坚定改革开放再出发的信心，破除体制机制障碍，优化营商环境，再创发展新优势。

加快重点领域改革。推进政府机构改革。按照中央、省统一部署，遵循优化职能、协同高效的原则，做好市县政府机构改革各项工作，3月底前基本完成改革任务。深化“放管服”改革。用互联网思维做政务，纵深推进“一网通办”前提下“最多跑一次”改革，加快全市一体化网上政务服务平台建设，让群众享受政务服务像网购一样便捷。加强实体政务大厅规范化建设，设置综合窗口，开展无差别受理服务，形成前台统一受理、后台分类审批、综合窗口出证的审批服务格局。建成“12345”政务服务热线平台。全面落实负面清单管理制度，优化审批流程，项目审批时限压减一半，让企业和群众切实感受到服务水平的提升。深化国企改革。推动市属国企建立现代企业制度，深化产权结构、组织结构、治理结构改革，引入战略合作者，盘活资产，做大做强。加强国有资产监管，定期向人大常委会报告监管情况，确保国有资产保值增值。深化“四医联动”综合医改，推进教育、科技等领域改革。

推动高水平开放。借鉴自贸区经验，制定我市“扩大对外开放行动计划”。主动融入“一带一路”，加快濮阳海关建设，力争4月底前正式开关，9月底前建成国际旅行卫生保健中心濮阳分中心，全面履行海关职能；提升公用型保税仓库功能，建设丰利石化油品保税罐区。参与“四条丝路”建设，对接郑州航空港、郑欧班列、天津港、日照港，构建空、铁、海联运格局，建设多式联运集散地。扩大濮阳至天津港“中原号”铁海联运班列业务，与天津港集团合作共建“无水港”，打造内陆开放节点城市。扩大开放招商，建立专业队伍，实施专业化、精准化招商。围绕主导产业，聚焦重点领域、重点区域、重点行业和重点企业，引进投资规模大、带动能力强、产业关联度高的优质项目。办好第六届中原（濮阳）油气技术装备展览会、2019中国涂料大会等招商活动。新签约、新开工亿元以上项目220个、160个，利用省外资金增速保持全省前列。加强区域合作，深化与菏泽、聊城战略合作，加强豫北区域合作，打造中原城市群北部跨区域协同发展示范区。与中原、京津冀、山东半岛三大城市群互动发展，对接发达地区资本、技术、项目，建设产业园区，发展“飞地经济”。

打造一流营商环境。营商环境是濮阳高质量发展的生命线，我们将打好“组合拳”，向一切破坏营商环境的行为宣战。建立健全以提供贴心服务为重点的亲商机制、以严打经济犯罪为抓手的护商机制、以落实各类政策为基础的惠商机制、以优化公共服务为平台的安商机制、以供给优质要素为保障的富商机制。开展营商环境第三方评估，建立完善营商环境评价指标体系，对营商环境建设成效开展年度测评。将营商环境建设纳入县区、市直职能部门绩效考评，明确考核细则，加大考核权重。规范执法行为，杜绝多头执法、重复执法、任性执法，让企业安心生产、放心经营。更加重视、支持民营企业发展，激发市场主体活力，让营商环境成为濮阳发展的新标识。

推进科技创新。构建开放、协同、高效的共性技术研发平台，健全以需求为导向、企业为主体的创新激励机制。重点培育国家高新技术企业、国家科技型中小企业、市级及以上创新龙头企业和“科技小巨人”企业34家。新建10家市级及以上工程技术研究中心，3家市级重点实验室，新增3家市级农业科技园区，新建1个院士工作站。推进中科院河南产业技术创新与育成中心濮阳分中心建设，支持蔚林新材料与清华大学合作共建智能化工研究院。加快开发区、濮东产业集聚区两个省级专利导航产业发展实验区建设。继续举办中国濮阳石化产业融合发展大会。力争专利权质押贷款8000万元，新增科技贷款3000万元，推动科技与金融有机结合。坚持人才强市，柔性引进30名以上高层人才，新增1家省级创新型科技团队。出台更加开放、优惠、务实的政策，政治上给荣誉、待遇上给实惠、事业上给舞台，让各类人才在濮阳有奔头、有干头。

（七）保障改善民生，增进人民福祉。围绕人民群众关切，集中更多资源投向民生领域，让发展更有“温度”，让幸福更有“质感”。

办好民生实事。优化财政支出结构，严控和压缩一般性支出，提高民生保障水平。一是新增城镇就业4.24万人。二是提高高龄老人医保报销比例，继续执行高龄津贴补助制度。三是继续免费开展妇女宫颈癌和乳腺癌筛查、预防出生缺陷产前筛查和新生儿疾病筛查。实施残疾儿童康复救助计划，全年救助658名残疾儿童。四是完善农村基础设施。完成70%农村户用卫生厕所改造，实施120个行政村农村人居环境整治工程；新建90公里农村公路，改扩建270公里，实施村村通客车提质工程，通车率达到100%；完成150个行政村电网改造升级；建设718个基层综合性文化服务中心。五是持续改善大气环境质量，全市空气质量主要指标PM2.5、PM10、优良天数完成省定目标任务。六是新建21所、改扩建12所城乡幼儿园，新增6870个学位；新建13所、改扩建7所城区中小学，新增41545个学位；建成37所农村标准化寄宿制学校。七是继续推进供暖工程。全市新增集中供暖面积556万平方米，完成“电代煤、气代煤”用户13.88万户，新增地热能供暖面积187万平方米，建筑能效改造提升541万平方米。八是加快县人民医院提质升级，二级甲等以上县级人民医院再增加1至2所。九是实施丹江水进农村工程。让清丰县、南乐县两县20个乡镇516个村79.8万人喝上丹江水。十是推进黄河滩区居民迁建。完成二批试点迁建任务，加快推进9个安置区建设，确保年内搬迁群众8000户以上。民生问题无小事，群众利益大于天。我们将以“钉钉子”精神，一件一件抓落实，说到做到、做就做好，努力让人民群众生活得更舒心、更幸福。

加强就业社保。抓好高校毕业生、退役军人等重点群体就业。建设职业培训和技能人才队伍建设示范城市，完成各类职业技能培训12万人次。新增农村劳动力转移就业1.2万人。提高城乡最低生活保障标准和补助水平，城乡居民基础养老金最低标准提高到105元。持续提高城乡居民基本医保补助标准，完善大病保险、困难群众大病补充医疗保险和医疗救助制度。持续推进残疾人精准康复服务行动。坚持“房子是用来住的，不是用来炒的”定位，完善住房市场体系和保障体系，探索共有产权房建设，更好解决群众住房问题。

优先发展教育。推动城乡教育均衡发展，争取濮阳县创成国家义务教育发展基本均衡县。优化城区中小学校布局，均衡配置优质教育资源，坚决整治“择校乱象”，着力解决大班额问题。加强学校周边租赁房治理。用好国家优质普惠学前教育扩容工程试点市机遇，开展城镇小区配套幼儿园治理工作，促进学前教育普及普惠发展。深入开展校企合作，大力发展职业教育。制定河南大学濮阳工学院发展规划，增设专业，扩大规模，逐步实现本科教育独立办学。濮阳医专拓展6个专业，建设附属医院，提高办学水平。支持濮阳技师学院创建国家级重点技师学院。

发展文化事业。全面加强精神文明建设，践行社会主义核心价值观，大力弘扬焦裕禄精神、红旗渠精神、愚公移山精神和“三李”精神，实施文化惠民工程，提升全社会文明程度和全民文明素质。优化网络舆论生态，打造清朗网络空间。完善公共文化服务体系，保护非物质文化遗产，建设市博物馆，提升市美术馆服务职能，加强县区“三馆一站”建设，提高基本公共文化服务覆盖面。

建设健康濮阳。加强公共卫生服务体系建设，实行分级诊疗制度，推进医疗联合体建设和远程医疗服务。加快推进电子健康卡项目，力争实现基层医疗机构电子健康卡全覆盖，二级以上医院信息互联互通、资源共享。推进国家医养结合试点城市建设。建成市人民医院综合病房楼，加快建设市妇幼保健院妇幼综合楼，启动实施市中医院骨科医院病房楼项目。县级医院14个项目基本建成。加强与知名医院合作，打造特色专科，为建设区域医疗中心创造条件。关心青少年身心健康，加强青少年近视防护。开工建设全民健身综合馆、游泳馆、新体校，积极创建全国全民健身模范市。

创新社会治理。加快信息化、立体化社会治安防控体系建设，推进“雪亮工程”全覆盖。深入开展扫黑除恶专项斗争，依法打击各类违法犯罪活动，严打严防严管严治。创建全国禁毒示范城市，深化禁毒人民战争。以创建国家食品安全示范城市为抓手，创新监管方式，确保人民群众舌尖上的安全。坚持发展新时代“枫桥经验”，妥善化解各类矛盾纠纷。推动社会治理重心下移，加强社会心理服务体系建设，实现政府治理和社会调节、居民自治良性互动，确保社会大局和谐稳定。

深化油地合作。油地一家人、油地一家亲，建设幸福美好家园是油地共同的责任。坚持油地高层联席会议制度，拓宽合作领域，提升合作层次，携手推进国家战略储气库群等重大项目建设，支持油田稳定产量、挖潜增效。做好“四供一业”移交后续工作，投资19.7亿元，对道路、供热、供水、供气、供电、污水处理等基础设施进行改造提升，确保交得出、接得住、管得好，让广大油田职工家属享受优质高效的公共服务。加强与中石化、河南能化等央企省企合作，共同推进原料结构调整、煤制合成气等产业项目，实现共赢共荣。

加强国防教育和国防后备力量建设，支持驻濮解放军、武警部队、预备役部队、消防救援队伍、民兵和人民防空建设，深入开展双拥共建，巩固军政军民团结。全面贯彻党的民族政策和宗教工作基本方针，促进民族团结、宗教和睦。支持工会、共青团、妇联等人民团体更好发挥桥梁纽带作用。加强统计工作，扎实做好第四次经济普查。统筹做好发展研究、机关事务、驻外联络、退役军人事务、公积金管理、史志、气象、邮政、通信等工作。

四、建设人民满意的政府

新时代新任务对政府自身能力提出了新要求。各级政府必须切实转变职能，担当作为，锐意进取，营造学的氛围、严的氛围、干的氛围，为人民提供优质高效服务。

突出政治引领。以党的政治建设为统领，把党的政治建设贯穿政府工作各领域、全过程。全面学习贯彻习近平新时代中国特色社会主义思想，往深里走、往实里走、往心里走，学懂弄通做实，树牢“四个意识”、坚定“四个自信”、做到“两个维护”，悟出坚定信仰，学到忠诚担当，内化于心、外化于行，在政治立场、政治方向、政治原则、政治道路上同以习近平同志为核心的党中央保持高度一致。对于习近平总书记重要指示批示和党中央大政方针，坚决做到贯彻落实不走样，决不允许不敬畏、不在乎、喊口号、装样子。对于省委、省政府和市委决策部署，坚决做到贯彻落实不变调，确保政令畅通、令行禁止。

坚持依法行政。全面落实《法治政府建设实施纲要（2015—2020年）》，依法施政、依法行政、依法办事，建设职能科学、权责法定、执法严明、公开公正、廉洁高效、守法诚信的法治政府。严格按照法定权限和法定程序行使权力，善于运用法治思维和法治方式深化改革、推动发展、化解矛盾、维护稳定。依法决策，完善程序，发扬民主，提高政府决策科学化、民主化、法治化水平。推进政务公开，加强政策宣传解读，主动回应社会关切。信守承诺，决不能“新官不理旧账”。依法接受人大及其常委会监督，自觉接受政协民主监督和监察机关的监督，主动接受社会和舆论监督，强化审计监督。认真听取人大代表、政协委员意见，办理好人大代表建议和政协提案。广泛听取民主党派、工商联、无党派人士和各人民团体意见。做好“七五”普法工作。加强政府层级监督，做好行政复议和行政应诉工作。推进服务型行政执法建设，严格行政执法监督，防止任性用权，纠正粗暴执法，决不允许以权代法、以权压法、逐利违法、徇私枉法，让公平正义阳光普照人民群众。

强化实干担当。坚持为民情怀，始终心系人民，敢于为民担当，深入调查研究，倾听群众心声，关心百姓疾苦，倾心为民办事。坚持解放思想，破除路径依赖的惯性、破除小进即满的惰性、破除“坐井观天”的狭隘，树立全球视野、时代眼光，跳出濮阳看濮阳，积极抢抓机遇，大胆创造机遇，打开发展新天地。增强市场意识，按市场规律办事，善用市场思维、市场方法解决困难问题。坚持提升本领，加强学习，形成与岗位相匹配的政治素质、理论功底、专业素养，弥补知识弱项、能力短板、经验盲区，弘扬工匠精神，追求完美卓越，争创一流业绩。坚持斗争精神，提升斗争本领，砥砺斗争意志，不回避矛盾，不推卸责任，迎难而上，敢啃硬骨头，善打攻坚战。狠刹“文山会海”，坚决杜绝各种形式主义、官僚主义，腾出更多精力推动工作、抓好落实。

打造廉洁政府。扛牢政府系统全面从严治党主体责任，认真履行“一岗双责”，全面加强党风廉政建设。锲而不舍落实中央八项规定及实施细则精神，坚决抵制“四风”新动向新表现。坚决惩治腐败，巩固发展压倒性胜利，紧盯重大工程、重点领域、关键岗位，加大反腐力度，发现一起、查处一起。严肃查处群众身边不正之风和腐败问题。坚持标本兼治，完善不敢腐、不能腐、不想腐长效机制，做到干部清正、政府清廉、政治清明。

各位代表！实干成就梦想，奋斗赢得未来。濮阳发展与每个濮阳人的命运息息相关，濮阳出彩与每个濮阳人的荣光紧紧相连，我们要强化家园意识，发扬主人翁精神，植根龙都大地，矢志干事创业，争做担当濮阳人、出彩濮阳人。让我们更加紧密地团结在以习近平同志为核心的党中央周围，高举习近平新时代中国特色社会主义思想伟大旗帜，在省委、省政府和市委的坚强领导下，初心不改、驰而不息，加快建设富裕文明和谐美丽新濮阳，阔步走向中原城市群改革开放前沿，为中原更加出彩增色添彩，以优异成绩庆祝中华人民共和国成立70周年！
